# Pan-cancer analysis of Homeobox B9 as a predictor for prognosis and immunotherapy in human tumors

**DOI:** 10.18632/aging.204785

**Published:** 2023-06-09

**Authors:** Qingdong Jin, Li Xu, Jun Wang, Junling Lin, Huang Lin

**Affiliations:** 1 The School of Clinical Medicine, Fujian Medical University, Fuzhou, Fujian Province, China; 2 Department of Neurosurgery, The First Hospital of Putian City, Putian, Fujian Province, China; 3 Key Laboratory of Translational Tumor Medicine in Fujian Province, Putian University, Putian, Fujian Province, China

**Keywords:** neurology, cancer

## Abstract

Background: Although several animal and cell studies have described the association between HOXB9 and cancers, there is no pan-cancer investigation of HOXB9. In this article, we explored the expression levels and prognosis of HOXB9 in pan-cancer. We evaluated the correlation of HOXB9 expression level with the efficacy of immunotherapy.

Methods: We conducted a survival analysis of HOXB9 in various types of cancer using publicly available databases. We also examined the relationship between HOXB9 expression levels and several factors including prognosis, immune infiltration, immune checkpoint genes, tumor mutational burden, microsatellite instability, mismatch repair, and DNA methylation. TIMER2.0 tool was conducted to explore the immune cell infiltrations related to HOXB9 in this analysis.

Results: It was discovered through a comprehensive analysis of multiple public datasets that HOXB9 expression was highly expressed in most tumor tissues and cancer cell lines and that distinct associations exist between HOXB9 expression and tumor patient prognosis. Besides, HOXB9 expression was closely associated with immune cell infiltration and checkpoint genes in many cancers. Further, HOXB9 was associated with immune cell infiltration, TMB, MSI, MMR, and DNA methylation. It was also confirmed that HOXB9 was highly expressed in clinical GBM tissues. Experiments further revealed that knockdown of HOXB9 expression could suppress proliferation, migration, and invasion of glioma cells.

Conclusions: The results revealed that HOXB9, a robust tumor biomarker, has a significant prognostic value. HOXB9 may act as a new predictor to assess cancer prognosis and therapeutic efficacy of the immune in various cancers.

## INTRODUCTION

In the past few decades, there has been a consistent decrease in tumor mortality, which can be attributed to the progress made in the field of cancer treatment, as stated by the American Cancer Society [1]. Although the number of cancer patients who pass away each year continues to rise, there has been a notable improvement in the treatment of various cancers through the use of immune checkpoint inhibitors like cytotoxic T lymphocyte-associated protein 4 (CTLA-4) and programmed cell death protein-1 (PD-1). These inhibitors have been increasingly used in immunotherapy in recent years and have played a significant role in the fight against cancer [2, 3]. Despite the availability of various antigens for cancer treatment, a significant proportion of cancer patients still exhibit resistance to these treatments. It was not possible to achieve long-lasting responses [4]. Thus, the search for new immunotherapy biomarkers or immunoregulatory genes is crucial in developing more precise immunotherapy schemes that can improve the durability of immune responses in cancer patients.

The HOX genes are a group of transcription factors that contain a highly conserved Homeobox domain. These genes are found in both invertebrates and vertebrates, but in invertebrates, there are 39 HOX genes that are grouped into four clusters, which are named HOXA, HOXB, HOXC, and HOXD [5, 6]. HOX factors regulate cell proliferation, invasion, and migration abilities [7]. Recent studies have shown that abnormal HOX gene expression can lead to malignancies, including lung, ovarian, prostate, and breast carcinoma [8–10], and interaction between HOX genes and the WNT/β-catenin signaling pathway has been involved in the development and progression of tumors [11–13].

The Homeobox B9 gene belonged to the HOX family and was implicated in molecular regulatory processes in many cancers [14, 15]. Specifically, numerous pieces of evidence have suggested that HOXB9 is involved in tumorigenesis and progression by promoting cell proliferation, invasion, and metastasis. Increased expression of HOXB9 has been linked to unfavorable survival outcomes in multiple types of cancer, such as adrenocortical carcinoma [16], lung adenocarcinomas [14, 17, 18], and breast cancer [19, 20]. It is reported that Homeobox B9 is overexpressed in liver cancer and colorectal cancer and promotes tumor cell progression [21, 22]. Moreover, Carbone et al. [23] found a strong correlation between HOXB9 and resistance to vascular endothelial growth factor inhibitor treatment in patients with colorectal cancer. The TGF-β signaling pathway regulates HOXB9 expression and triggers the activation of EMT, which is associated with increased angiogenesis, lung metastasis, and radioresistance in cancer cells [19, 24]. HOXB9 has been shown to drive the progression of endometrial cancer by targeting E2F3 [25]. HOXB9 inhibits the proliferation of pancreatic cancer cells by blocking cell cycle progression through the DNMT1/RBL2/c-Myc axis [26]. HOXB9 was also an enabler of multi-organ metastasis of lung adenocarcinoma by activating the WNT/TCF pathway and was also a metastatic promoter of colon cancer and a potential biomarker of bevacizumab therapy [17].

To summarize, HOXB9 has been extensively studied in relation to cancer progression and is considered a crucial player in this process. However, its impact on cancer immune infiltration and prediction of immunotherapy response remains unclear, and there has been no comprehensive investigation of its role across different types of cancer. The first step of our study involved analyzing multiple databases to investigate the expression levels and clinical significance of HOXB9 in 33 different types of cancer. We reveal the association of HOXB9 with evaluation indicators of pan-cancer, such as immune infiltration, immune checkpoint genes (ICGs), TMB, MSI, MMR, and DNA methylation in pan-cancer. Furthermore, HOXB9 was confirmed to be a potent oncogene in glioma cell line experiment. Overall, our findings confirmed that HOXB9 is a robust prognostic biomarker for various types of cancer and can effectively predict the response to immunotherapy. These results serve as a foundation for further exploration into the role of HOXB9 in cancer immunity. Thus, this study represents the first attempt to comprehensively elucidate the tumorigenic role of HOXB9 across various types of cancer, and it offers valuable insights that can guide future research on HOXB9. These findings hold promise for advancing our understanding of HOXB9’s impact on cancer development and progression and may inform the development of novel therapeutic strategies targeting this gene.

## MATERIALS AND METHODS

### Data collection and processing

To fully understand the expression patterns of HOXB9, we collected multiple different public datasets, including the TCGA [27], CCLE [28], and GTEx databases [29]. We obtained HOXB9 expression data from the GTEx program for 31 normal tissues and from the CCLE database for 21 tumor cell lines. We retrieved data on HOXB9 expression levels in both cancer and normal tissues from the TCGA pan-cancer cohort and GTEx datasets via the UCSC Xena database. Additionally, the relationship between HOXB9 expression and different pathological stages (stage I-IV) of TCGA tumors via the GEPIA2 website (http://gepia2.cancer-pku.cn/#analysis). Also, the mutation levels of MMR genes and DNA methyltransferases of different types were analyzed from the TCGA database. Pearson correlation analysis was used to further evaluate the associations between HOXB9 and methyltransferases. Immunofluorescence data of HOXB9 in different cells were obtained from the THPA database (https://www.proteinatlas.org/). The abbreviations of cancers were represented in Supplementary Table 1.

### Genetic alteration analysis

The cBioPortal for Cancer Genomics web tool was employed to examine the frequency of genomic alterations affecting HOXB9. After logging into the cBioPortal web (https://www.cbioportal.org/) [30, 31], we observed HOXB9 alteration frequency, mutation type, and Copy number alteration outcomes in all TCGA tumors. In addition, the distribution and 3D structure of the mutated site of HOXB9 were also displayed in the diagram.

### Gene enrichment analysis of HOXB9-related partners

Using the datasets from TCGA for both tumors and normal tissues, we utilized the “Similar Gene Detection” function of GEPIA2 to identify genes that are correlated with HOXB9. HOXB9 and selected genes were also subjected to a pairwise Pearson correlation analysis using GEPIA2’s correlation analysis module. The log2 TPM was used in the dot plot. The resulting analysis showed both the correlation coefficient (R) and the associated P-value for each of the identified genes.

### Prognosis analysis of HOXB9 in pan-cancer

We collected prognostic information on four distinct types of outcomes from the UCSC Xena. The prognostic significance of HOXB9 for a specific type of prognosis in each cancer was evaluated using both univariate Cox regression and the Kaplan-Meier model. We generated a heatmap to visualize the outcomes, which included the log-rank p-value calculated using the Kaplan-Meier (K-M) method, as well as the hazard ratio (HR) with a 95% confidence interval (95% CI).

### Single-cell analysis of HOXB9

We utilized the Tumor Immune Single-cell Hub (TISCH) web tool to conduct a single-cell analysis related to our study. This tool allows for the exploration of tumor and immune cell heterogeneity in various cancer types at the single-cell level (http://tisch.comp-genomics.org/documentation/) [32]. Specifically, we used the following parameters: HOXB9 as the gene of interest, major-lineage as the cell-type annotation, and all cancers as the cancer type. Heatmap allows us to visualize and quantify the HOXB9 expression levels for every kind of cancer.

### Gene set enrichment analysis

We downloaded the “gmt” file for the hallmark gene set from the Molecular Signatures Database website (https://www.gsea-msigdb.org/gsea/index.jsp). This file contains 50 different gene sets associated with hallmark biological processes. To identify differentially expressed genes between the low- and high-HOXB9 levels cancer groups in each cancer type, we utilized the hallmark gene set. We calculated the normalized enrichment score (NES) and false discovery rate (FDR) for each biological process using this gene set. The GSEA was conducted by taking the R package “clusterProfiler” [33], we summarized and visualized the results using a bubble plot.

### Immune cell infiltration enrichment

TIMER is a web-based tool that provides a user-friendly interface for quantifying immune cell infiltration across different cancer types. The TIMER2 database (http://timer.cistrome.org/) was used to obtain data on immune cell infiltration levels in TCGA cancers. We analyzed the expression of HOXB9 in different cancer types. HOXB9 expression of various cancers was analyzed, and the relationship between HOXB9 and different immune infiltrates was detected by Spearman correlation analysis.

### Immunotherapy prediction analysis

To evaluate the statistical correlations between HOXB9 and established immunotherapy biomarkers such as TMB, MSI, and immune checkpoint genes in pan-cancer, Spearman correlation analysis was performed. We obtained multiple cohorts of patients who had received immune checkpoint blockade therapy to validate whether HOXB9 could predict the response to this type of immunotherapy. HOXB9 expression and the immunotherapeutic effect were obtained from GSE91061 [34], Gide2019 [35], and the Vanallen2015 cohort [36]. The GSE91061 cohort refers to a collection of transcriptomic profiles from 51 melanoma patients who were treated with anti-PD1 (nivolumab). The purpose of this cohort is likely to study the gene expression patterns in response to this specific treatment in order to identify potential biomarkers or therapeutic targets for melanoma. The Gide2109 cohort includes 32 melanoma patients’ transcriptomic profiles receiving combined anti-PD-1 and anti-CTLA-4 immunotherapy. The Vaballen2015 cohort contains 42 melanoma patients treated with anti-CTLA-4 (atezolizumab).

### Specimen collection

For this study, we obtained samples of glioblastoma multiforme (GBM) and adjacent tissues from inpatients receiving treatment in the Neurosurgery Department of The Putian First Hospital. Before collecting any samples, informed consent was obtained from all the patients who were admitted to the hospital and enrolled in the study. The clinical sample collection and usage processes strictly followed the guideline. After excising from patients, tumor specimens were preserved in liquid nitrogen. The Medical Ethics Committee of Putian First Hospital approved this study.

### Cell lines and reagents

We obtained the SW1783, SW1088, and U87 cell lines, as well as Normal Human Astrocytes (NHA), from the Chinese Academia Sinica Cell Repository. These cell lines underwent authentication through STR profiling to ensure their identity and purity. Standard cell culture techniques were employed to culture the cells using Dulbecco’s Modified Eagle’s Medium (DMEM) from Gibco (USA), supplemented with 10% fetal bovine serum (FBS). Reagents are included in Supplementary Table 2.

### Transfection and Western blotting analysis

Plasmids encoding short hairpin RNAs (shRNA) against HOXB9 were synthesized by Genepharma Company (Shanghai, China). In a 6-well plate, cells were seeded at a density of 1×10^5^ cells/well. We followed the manufacturer’s instructions to transfect the cells with the shRNA plasmids and vectors, using Lipofectamine^™^ 3000. The western blotting assay was conducted following the conventional experimental procedures. Briefly, the protein was separated by SDS-PAGE and then transferred to nitrocellulose membranes**,** which were incubated with the primary rabbit polyclonal antibody of HOXB9 protein (1:300, SANTA CRUZ) and Alpha Tubulin Polyclonal antibody (1:1000, Proteintech) at 4° C overnight.

### Cell growth assays

Ethynyl-2-Deoxyuridine (EdU) Assay and Cell Counting Kit-8 (CCK-8) Assay were used to explore the cell proliferation ability. For CCK-8 assays, the cells were detected by a CCK8 kit (Beyotime, C0037, China) at 37° C based on the instructions provided by the manufacturer. To perform the EdU assay, we used the Cell-Light EdU Apollo EdU staining kit from Ribobio (Guangzhou, China) following the manufacturer's instructions. The assay was carried out on cells grown in 96-well plates until they reached an appropriate density. After completion of the assay, images were taken and the number of EdU-positive cells was counted.

### Transwell migration and invasion assays

Migration and invasion were taken in conventional experimental methods. Briefly, cells were resuspended in a serum-free medium and placed in the upper chambers. After the culture period, the cells that did not migrate and remained on the upper surface of the membrane were eliminated, while the cells that migrated and attached to the lower surface were fixed with paraformaldehyde for a duration of 30 minutes. The fixed cells were then stained with 0.1% crystal violet. We used an optical microscope to capture images and counted the cells in five random fields.

### RNA extraction and quantitative real-time PCR (qRT-PCR)

According to the instruction, we isolated total RNA from cells taking Trizol reagent (Invitrogen, USA). The cDNA was synthesized using the PrimeScript RT Reagent Kit (TaKaRa, Japan) for qRT-PCR. We used SYBR Green PCR Master Mix for quantitative analysis. GAPDH was taken as an internal control. The primer pairs used for the experiment can be found in the Supplementary Table 3. The fold changes were calculated using the ΔΔCT method.

### Statistical analysis

Differences between groups were analysed using two-tailed Student’s t-tests and analysis of variance (ANOVA). The correlation of HOXB9 expression with studied factors was performed by Spearman correlation analysis. The chi-square test was taken to determine the statistical significance of the proportions of ICI therapy responders and non-responders between the low- and high-HOXB9 cancer subgroups. The results were expressed as mean±SD from at least three independent experiments. All bioinformatics and statistical analyses were adopted using the R program and GraphPad Prism 9. The statistical significance of the data was determined when P < 0.05.

### Availability of data and materials

The main results are obtained using public datasets described in detail in this paper’s methodology. The names of the repository/repositories and accession number(s) can be found in the article. The remaining data can be obtained by contacting the corresponding authors with reasonable requirements.

## RESULTS

### Pan-cancer expression landscape of HOXB9

**Figure 1:**
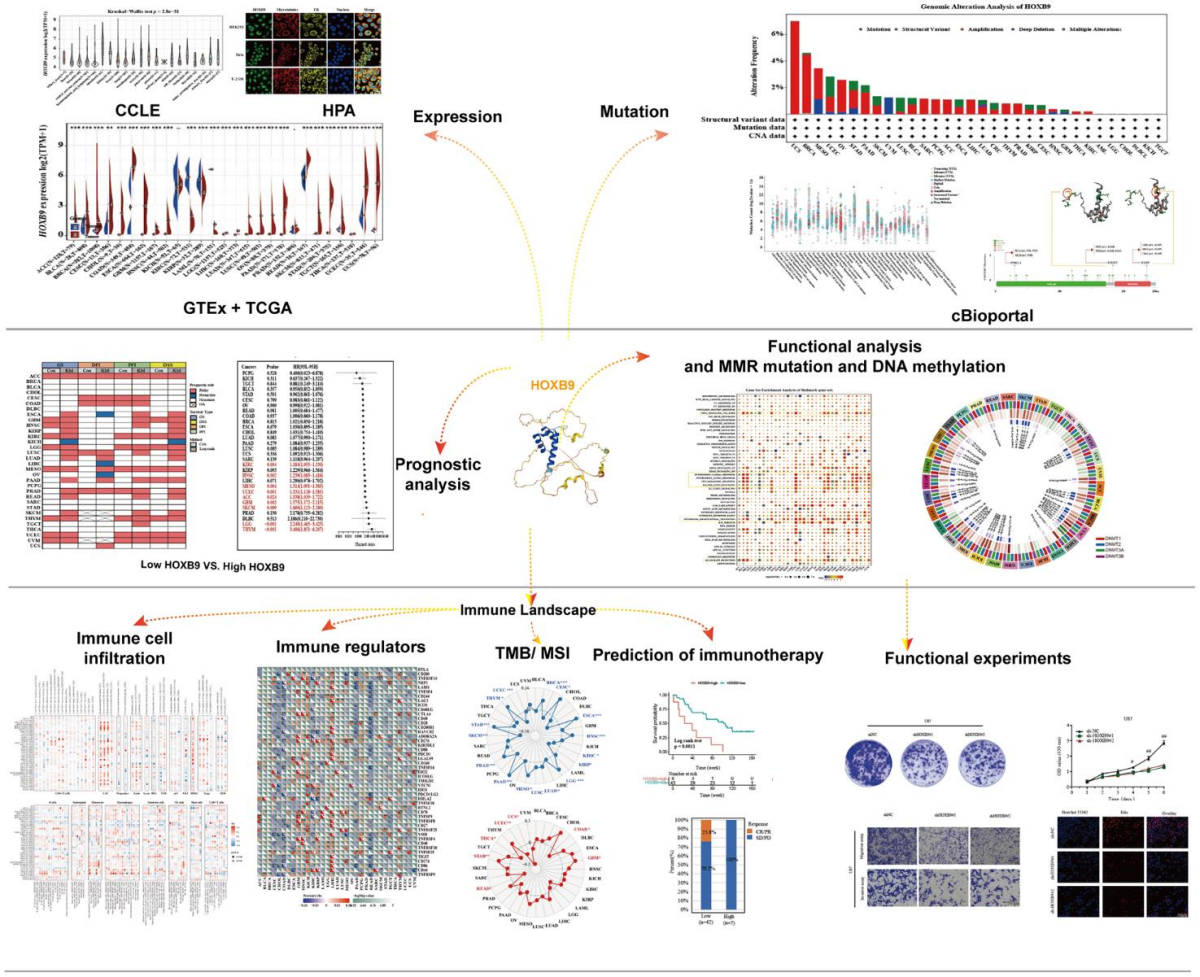
The flow chart of the entire study.

**Figure 2:**
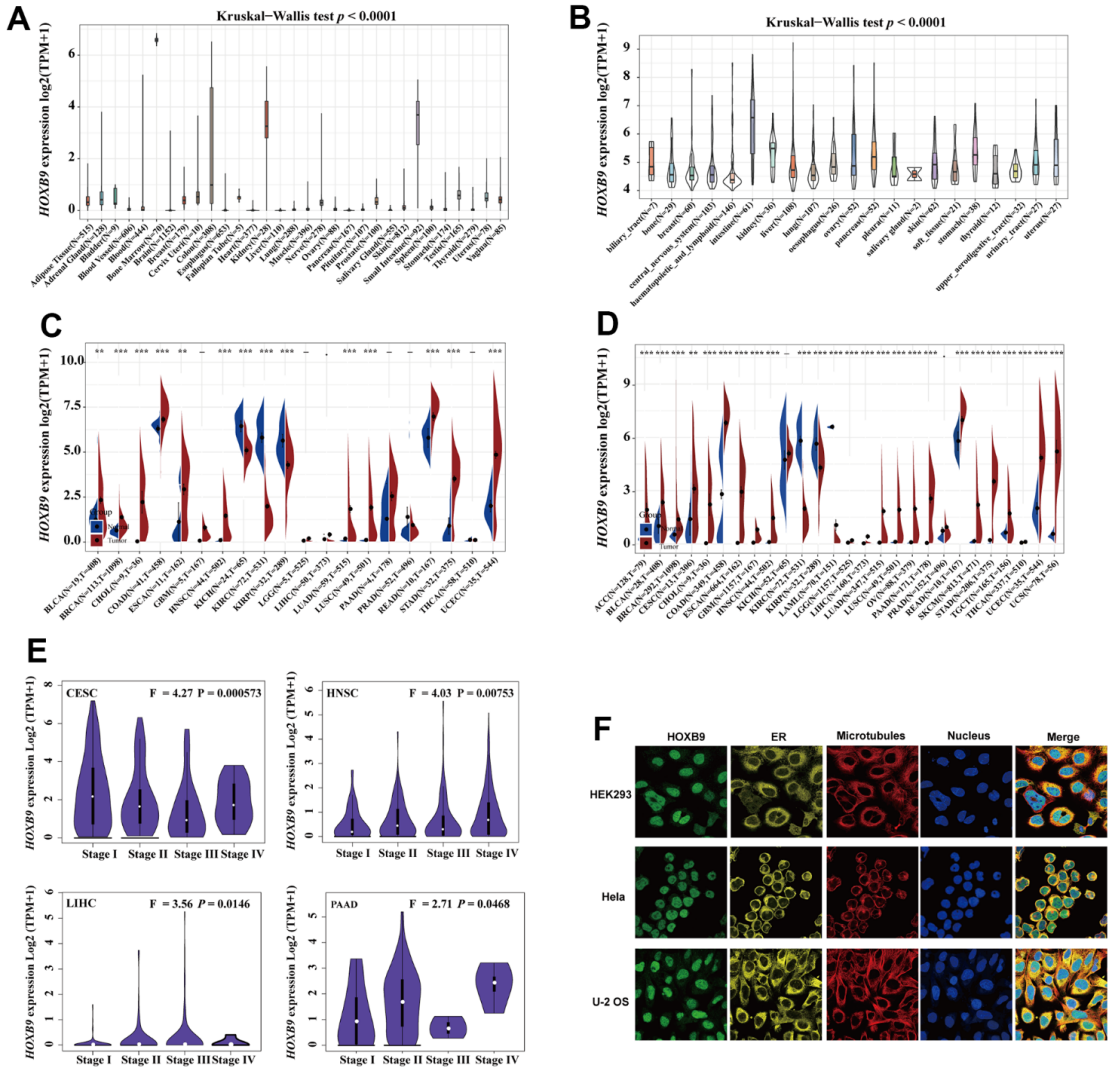
**HOXB9 expression in the human pan-cancer.** (**A**) The expression levels of HOXB9 in 31 diverse normal human tissues from the GTEx datasets. (**B**) The expression levels of HOXB9 in 21 diverse cancer cell lines from the CCLE database. (**C**) HOXB9 expression in cancers and normal tissues from TCGA database. (**D**) The expression levels of HOXB9 between tumor and normal tissues in each cancer are based on the integrated data from TCGA and GTEx datasets. (**E**) Based on the TCGA data, the expression levels of the HOXB9 gene were analyzed by the main pathological stages of CESC, HNSC, PAAD, and LIHC. (**F**) The immunofluorescence images of HOXB9 protein, nucleus, endoplasmic reticulum (ER), microtubules, and the merged images in HEK293, Hela, and U-2 OS cell lines. *P < 0.05, **P < 0.01, ***P < 0.001.

Figure 1 illustrated the study design in the form of a flow chart. To first characterize the basic information of HOXB9 in cancer, the GTEx database was applied to describe the levels of HOXB9 in normal tissues. The expression of HOXB9 varied significantly across different tissue types, with the greatest expression observed in bone marrow (Figure 2A). Furthermore, based on the CCLE data**,** we observed the HOXB9 expression in various cancer cell lines (Figure 2B). Moreover, compared with normal tissues, HOXB9 expression levels were relatively higher in BLCA, BRCA, CHOL, COAD, ESCA, HNSC, LUAD, LUSC, READ, STAD, and UCEC in the TCGA database (Figure 2C). Consider that in the TCGA database, for some types of cancer, the data of normal samples are much smaller than the data of tumor samples. We combined the TCGA and GTEx databases to examine the expression patterns of HOXB9 across various types of cancer. The results revealed that high expression levels of HOXB9 were examined in most cancers except for KICH, KIRP, KIRC, LAMAL, and PRAD (Figure 2D). Next, the significant relationship between the expression of HOXB9 and the pathological stages was found in the CESC, HNSC, PAAD, and LIHC (Figure 2E, *P* < 0.05) but not other cancers (Supplementary Figure 1). IF imaging revealed that the HOXB9 protein was localized in the nucleus in HEK293, Hela, and U-2 OS tumor cell lines (Figure 2F).

### Mutation feature of HOXB9 in different tumors of TCGA

**Figure 3:**
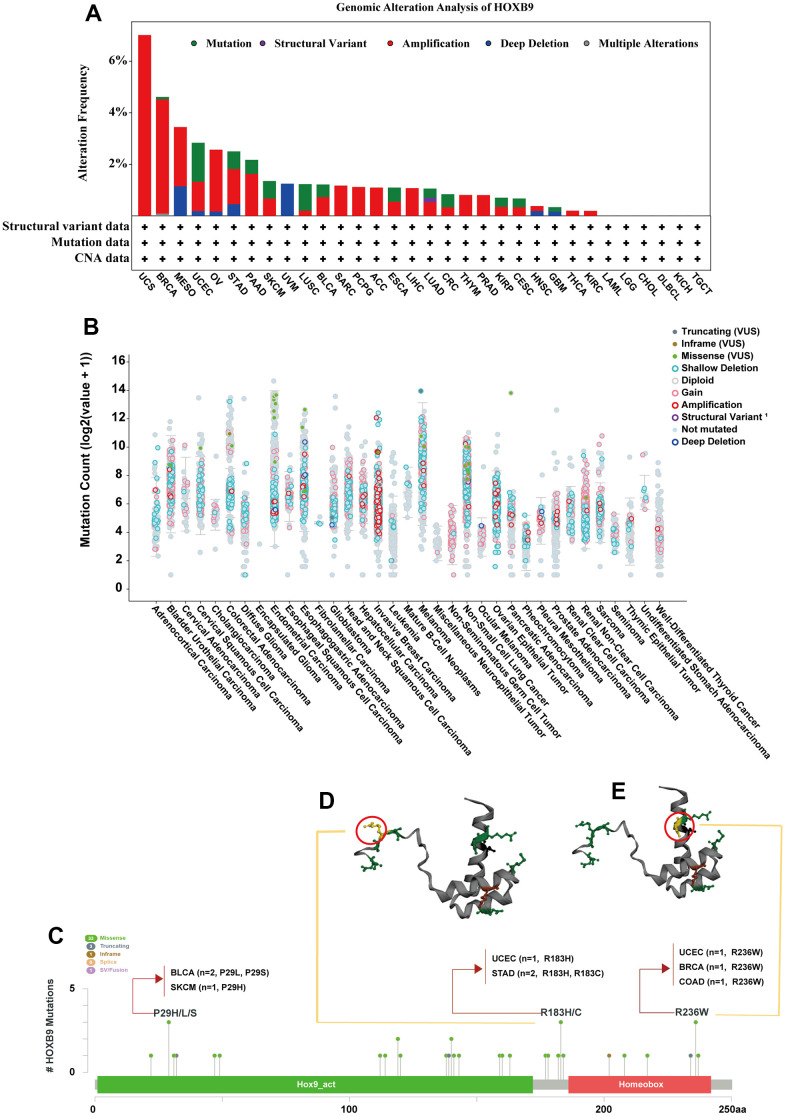
**Mutation feature of HOXB9 in different tumors.** (**A**) HOXB9 alteration frequency analysis in the pan-cancer study according to the cBioPortal database. The alteration included deep deletion (blue), structural variant (purple), multiple alterations (brown), amplifications (red), and mutations (green). (**B**) Mutation count of HOXB9 across all TCGA cancers using cBioPortal database. (**C**) The mutation sites of HOXB9 were determined according to the cBioPortal database. (**D**, **E**) The mutation site with the highest alteration frequency (R183H/C and R236W) in the 3D structure of HOXB9 was displayed.

We next analyzed the genetic alterations features of HOXB9 genes. HOXB9 genomic alteration analysis showed that alterations were widespread across multiple cancer types, and a total of 38 mutations were displayed in Supplementary Table 4. As shown in the Figure 3A, HOXB9 mutation frequencies were high in UCS, BRCA, MESO, UCEC, OV, STAD, and PAAD. Moreover, the cancer type with the highest frequency of HOXB9 alterations was UCS, exceeding 6% of UCS patients, and amplification. In addition, amplification is the primary type of HOXB9 variation in UCS, BRCA, OV, PAAD, SARC, PCPG, ACC, LIHC, THYM, PRAD, THCA, and KIRC. However, it is worth noting that the structural variant of HOXB9 was only present in LUAD patients. The mutation count of HOXB9 in various cancers was shown in Figure 3B. The types, sites, and cases of HOXB9 genetic mutation were demonstrated in the Figure 3C. In addition, the primary type of genetic alteration we found in HOXB9 was missense mutation, and P29H/L/S, R183H/C, and R236W alteration were detected in BLCA, SKCM, UCEC, STAD, BRCA, and COAD patients. We further display the 3D structure of HOXB9 of two-mutation sites with the highest alteration frequencies (R183H/C and R236W) (Figure 3D, 3E).

### Prognostic value of HOXB9 in pan-cancer

**Figure 4:**
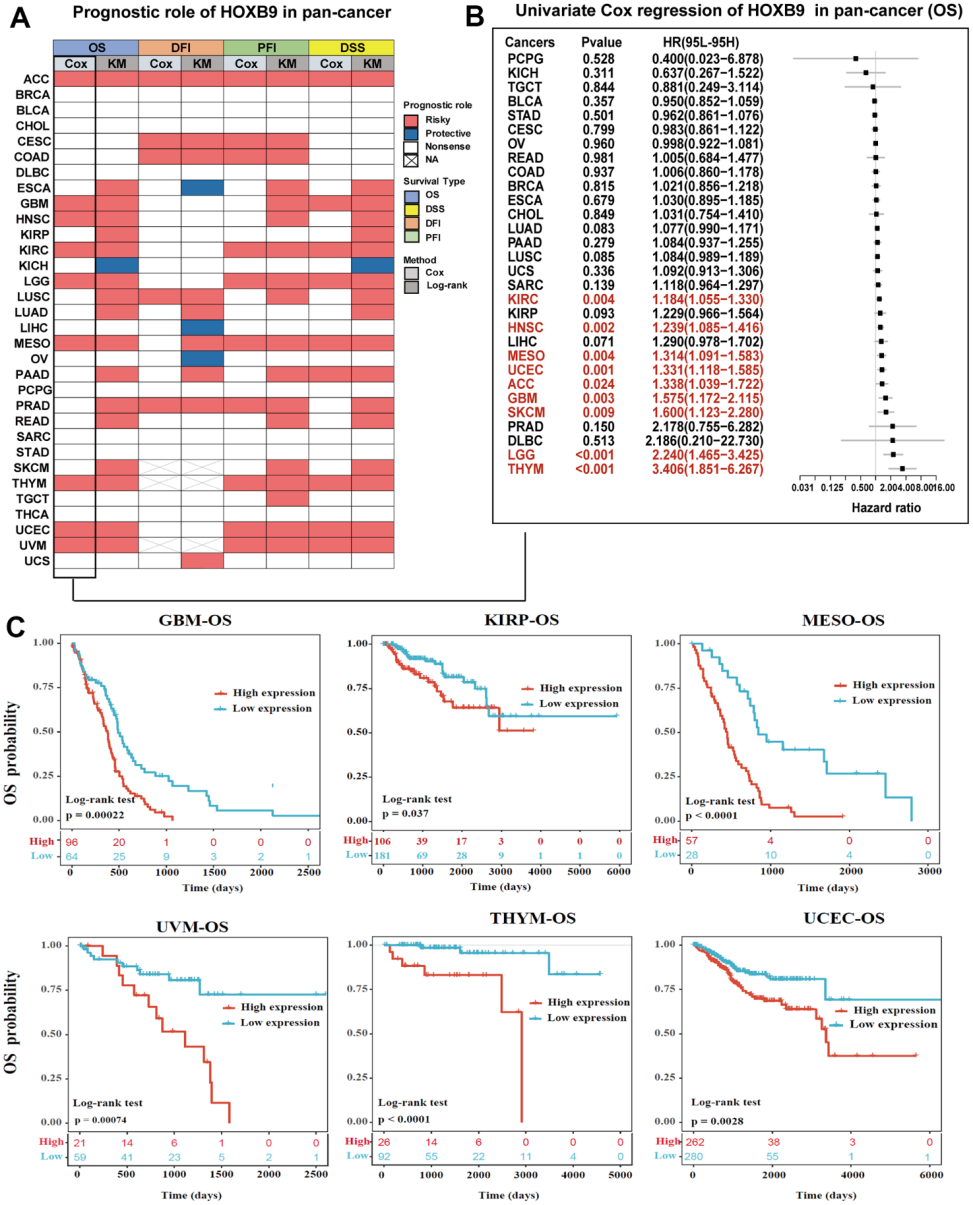
**Prognostic value of HOXB9 in pan-cancer.** (**A**) Summary of the correlation between expression of HOXB9 with overall survival (OS), disease-specific survival (DSS), disease-free interval (DFI), and progression-free interval (PFI) based on the univariate Cox regression and Kaplan-Meier models. Red indicates that HOXB9 is a risk factor affecting the prognosis of cancer patients, and blue represents a protective factor. (**B**) The forest map exhibited the prognostic role of HOXB9 in OS in cancers by the univariate Cox regression method. (**C**) Kaplan-Meier curves comparing overall survival probability in GBM, KIRP, MESO, UVM, THYM, and UCEC cohorts with high and low HOXB9 expression.

Further, we explored the prognostic value of HOXB9 for pan-cancer. The prognostic analysis heatmap revealed that the HOXB9 was correlated with the prognosis of most tumors, except for BRCA, BLCA, CHOL, DLBC, SARC, STAD, and THCA (Figure 4A). The OS analysis results indicated that HOXB9 is associated with poor prognosis for patients with ACC, ESCA, GBM, HNSC, KIRP, KIRC, LGG, LUSC, LUAD, MESO, PAAD, PRAD, READ, SKCM, THYM, UCEC, and UVM, while being a protective factor for patients with KICH. Furthermore, the DSS analysis showed a high consistency with the OS analysis, confirming that HOXB9 is a prognostic risk factor for the 17 cancers mentioned above. The prognostic impact of HOXB9 in various cancers was further assessed by examining disease-free interval (DFI) and progression-free interval (PFI) outcomes. The results of these analyses also demonstrated that most cancer types have identified HOXB9 as a risk factor. Notably, both OS and DSS analyses indicated that HOXB9 acts as a protective factor in KICH. Univariate Cox regression analysis in the TCGA dataset was conducted to study the effect of HOXB9 on patient prognosis. The forest plot analysis revealed that a decrease in HOXB9 expression was associated with a longer overall survival (OS) time in ACC (HR = 1.338 [95%CI, 1.039 to 1.722], *P* = 0.024), GBM (HR = 1.575 [95%CI, 1.172 to 2.115], *P* = 0.003), SKCM (HR = 1.600 [95%CI, 1.123 to 2.280], *P* = 0.009), LGG (HR = 2.240 [95%CI, 1.465 to 3.425], *P* < 0.001), THYM (HR = 3.406 [95%CI, 1.851 to 6.267], *P* < 0.001), UCEC (HR = 1.331 [95%CI, 1.118 to 1.585], *P* = 0.001), MESO (HR = 1.314 [95%CI, 1.091 to 1.583], *P* = 0.004), HNSC (HR = 1.239 [95%CI, 1.085 to 1.416], *P* = 0.002), KIRC (HR = 1.184 [95%CI, 1.055 to 1.330], *P* = 0.004) (Figure 4B). We performed Kaplan-Meier survival analysis to investigate the impact of HOXB9 on patient prognosis in different cancer types using patients’ data dichotomized for median gene expression level. The results showed that higher HOXB9 level was significantly associated with poor overall survival outcomes in GBM, KIRP, MESO, UVM, THYM, and UCEC (Figure 4C). Therefore, HOXB9 may be a prognostic biomarker of overall survival in the above cancers.

### Enrichment analysis of HOXB9-related partners

**Figure 5:**
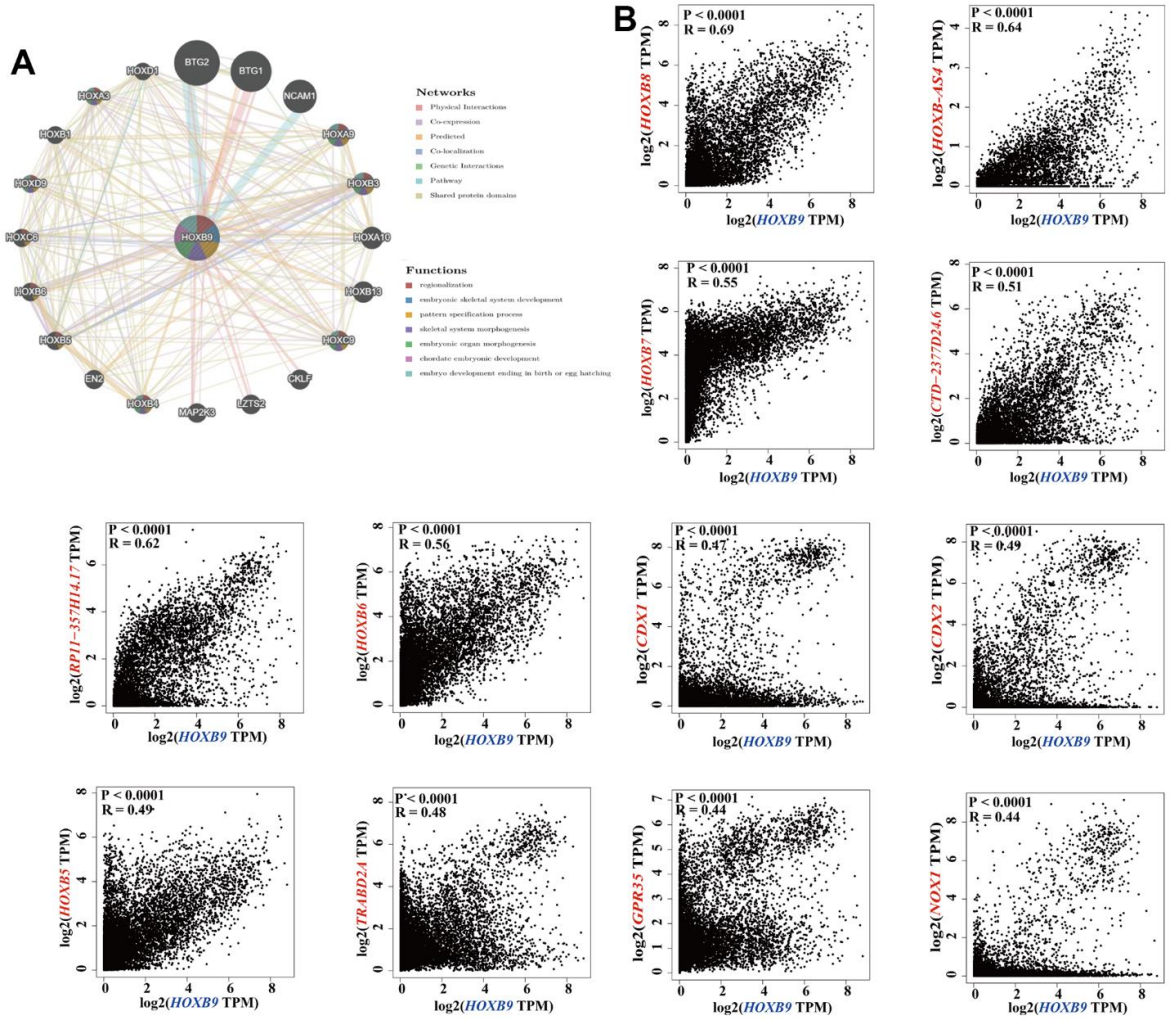
**HOXB9-related gene enrichment analysis.** (**A**) The protein-protein interaction (PPI) network presents the proteins interacting with HOXB9. (**B**) We also obtained the HOXB9-correlated genes in TCGA projects and analyzed the expression correlation between HOXB9 and selected targeting genes including HOXB8, HOXB-AS4, HOXB7, CTD-2377D24.6, RP11-357H14.17, HOXB6, CDX2, CDX1, HOXB5, TRABD2A, NOX1, GPR35 by the GEPIA2 approach.

To deeply analyze the possible underlying molecular mechanisms by which HOXB9 is involved in tumorigenesis, we attempted to identify HOXB9-binding proteins and genes that were correlated with HOXB9 expression in order to perform more comprehensive pathway enrichment analyses. Based on the GeneMANIA tool, we showed the interaction network of 20 HOXB9-related proteins (Figure 5A). Furthermore, we combined all TCGA tumor expression data with the GEPIA2 algorithm to find the major genes linked with HOXB9 expression. In the Figure 5B, the HOXB9 expression level was positively related to HOXB8 (Homeobox B8) (R = 0.69), HOXB-AS4 (R = 0.64), HOXB7 (Homeobox B7) (R = 0.55), CTD-2377D24.6 (R = 0.51), RP11-35H14.17 (R = 0.62), HOXB6 (Homeobox B6) (R = 0.56), CDX2 (Caudal Type Homeobox 2) (R = 0.49), CDX1 (Caudal Type Homeobox 1) (R = 0.47), HOXB5 (Homeobox B5) (R = 0.49), TRABD2A (TraB Domain Containing 2A) (R = 0.48), NOX1 (NADPH Oxidase 1) (R = 0.44), GPR35 (G Protein-Coupled Receptor 35) (R = 0.44) genes (all *P* < 0.0001).

### GSEA of HOXB9 in various cancer

**Figure 6:**
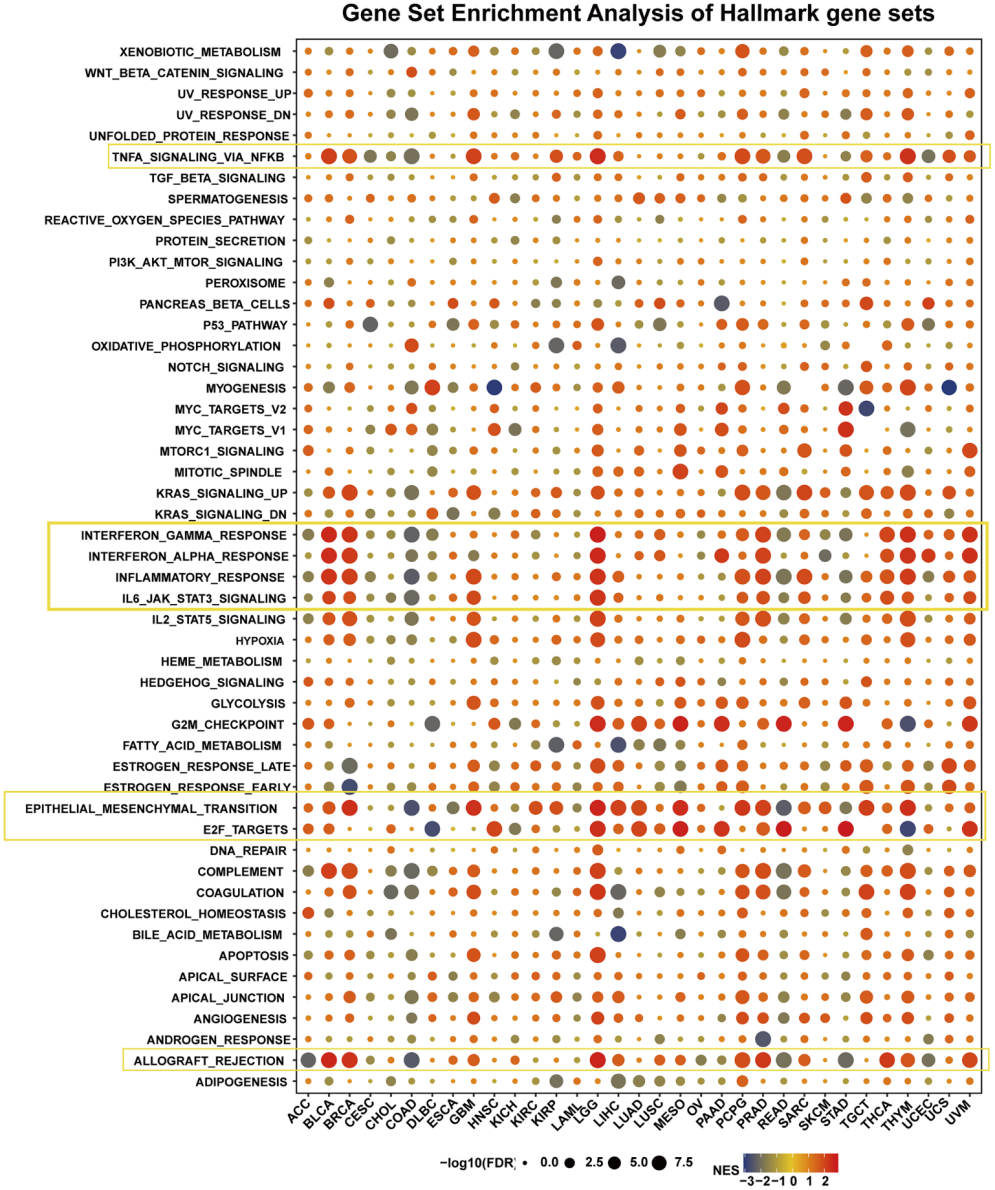
**The hallmarks gene set enrichment analysis of HOXB9 in cancers.** The circle size represents the FDR value of the enriching term in each tumor.

We assessed the pathway by which HOXB9 may be involved in GSEA in 33 tumors by taking the TCGA database. It is found that expression of HOXB9 was closely related to immune-related pathways, such as TNFA-signaling-via-NFKB, IFN-α/γ response, inflammatory-response, IL6-JAK-STAT3-signaling, and allograft-rejection pathways, especially in BLCA, BRCA, GBM, LGG, PCPG, PRAD, THCA, and THYM (Figure 6). We found a potential correlation between HOXB9 expression and immune activation in the tumor microenvironment. Furthermore, the high-HOXB9 subgroups showed a significant enrichment of the epithelial-mesenchymal transition (EMT) hallmark including BRCA, GBM, LGG, LIHC, LUAD, MESC, PCPG, PRAD, TGCT, and THYM. E2F-TARGETS signaling pathway was markedly negatively correlated with DLBC and THYM and positively correlated with HNSC, LGG, LUAD, MESO, PAAD, READ, STAD, and UVM. Besides, G2M checkpoint, complement, and coagulation were also tightly associated with HOXB9 expressions in some types of cancers, such as DLBC, LGG, LUAD, MESO, PAAD, READ, STAD, THYM, and UVM. To summarize, our data suggest that increased expression of HOXB9 is linked to immune activation in the tumor microenvironment and may offer insights for further investigation into the potential functions and impact of HOXB9 in cancer progression.

### TIMER immune cell infiltration analyses

**Figure 7:**
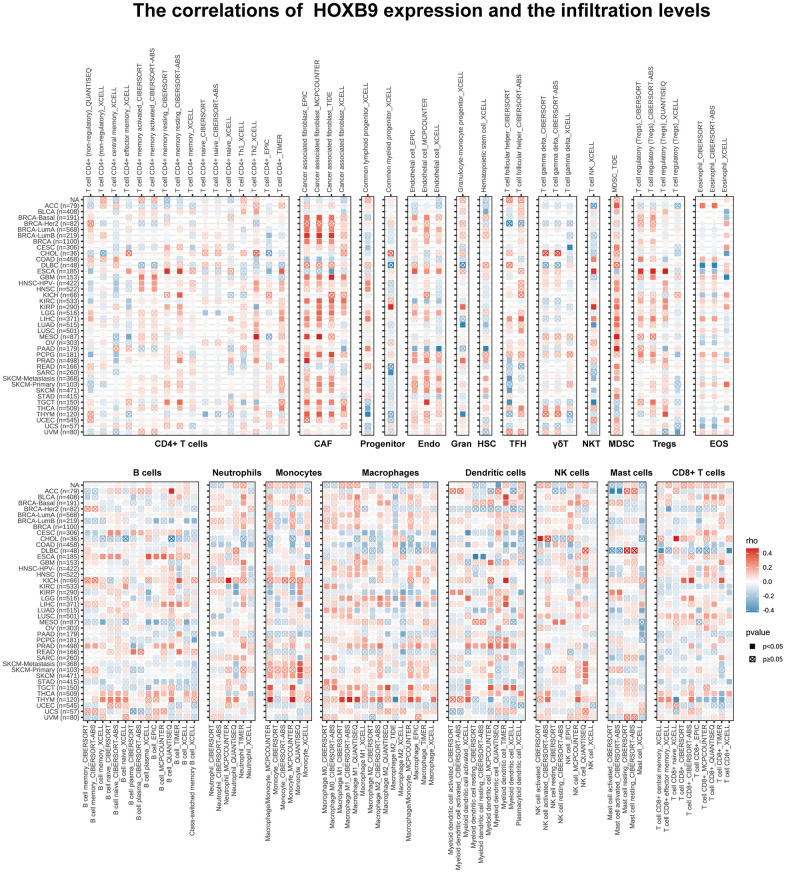
**Immune cell infiltration analysis.** The correlations of HOXB9 expression and the infiltration levels of CD4+ T cells, CAF, progenitor, Endo, Gran, HSC, TFH, γδT, NKT, MDSC, Tregs, EOS, B cells, Neutrophils, Macrophages, Dendritic cells, NK cells, Mast cells and CD8+ T cells in various cancers. Positive correlation in red and negative correlation in blue.

We further investigate the relationships between HOXB9 expression and immune cell infiltrations to demonstrate the links between HOXB9 and cancer immunity. We performed the Spearman correlation analysis using pan-cancer immune cell infiltration data. The infiltration levels in pan-cancer were shown in Figure 7. Our analysis demonstrated a positive correlation between HOXB9 expression and the infiltration levels of CAF, Endo, MDSC, macrophages, dendritic cells, and NK cells in most of the TCGA cancers. Moreover, in THYM, HOXB9 was positively related to the infiltration level of most immune cells such as CAF, B cells, monocytes, and macrophages. At the same time, it was negatively related to CD8+ T cells. Studies in recent years have shown that immune cell infiltration, such as CD4+T cells, CD8+ T cells, MDSC, CAF, etc., plays a crucial role in cancer immunotherapy [37, 38]. Hence, it is crucial not to overlook the significance of immune cells in cancer therapy. We used TISCH at the single-cell level to analyze the pan-cancer expression pattern of HOXB9. HOXB9 had relatively high expression in the myofibroblasts, endothelial, and fibroblasts, especially in myofibroblasts (Supplementary Figure 2). Lastly, the integration of ImmuneScore, EstimateScore, StromalScore, and neoantigens led to the observation that HOXB9 is linked to immune infiltration in certain types of cancer (Supplementary Figures 3–6). Overall, these findings strongly suggest that HOXB9 may have a significant impact on the cancers by interacting with immune cells.

### Correlation between HOXB9 expression and immune regulators, TMB, and MSI

**Figure 8:**
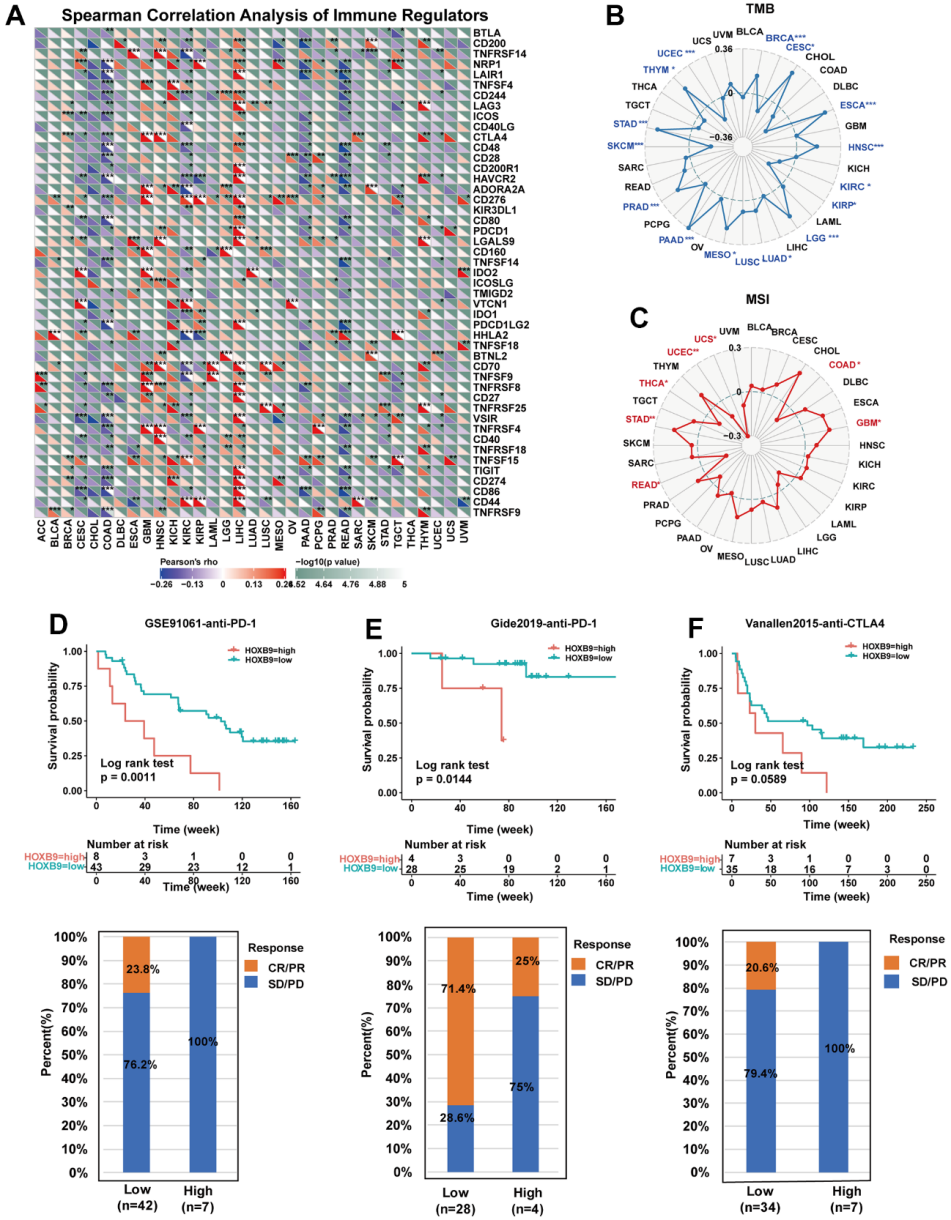
**Correlation between HOXB9 and immune regulators, TMB, and MSI.** (**A**) The Spearman correlation heatmap depicts the correlations between the HOXB9 expressions and the different types of immune regulators in pan-cancer. A positive correlation is represented by red, while a negative correlation is represented by blue. (**B**) Radar map of the correlation between HOXB9 expression and the TMB in pan-cancer. (**C**) Radar map of the correlation between HOXB9 expression and MSI in pan-cancer. (**D**) Kaplan-Meier curves for low- and high-HOXB9 patient groups in GSE91061 (anti-PD-1 therapy), and the fraction of melanoma patients responding to anti-PD-1 therapy in low- and high-HOXB9 subgroups of GSE91061. (**E**) Kaplan-Meier curves for low- and high-HOXB9 patient groups in the Gide2019 cohort (anti-PD-1 and anti-CTLA4 therapy), and the fraction of melanoma tumors patients with response to a combination of anti-PD-1 and anti-CTLA4 therapy in low- and high-HOXB9 subgroups of Gide2019 cohort. (**F**) Kaplan-Meier curves for low- and high-HOXB9 expression groups from the Vanallen2015 cohort receiving anti-CTLA4 immunotherapy, and proportion of patients with therapeutic response to anti-CTLA4 blockade immunotherapy in low- and high-HOXB9 expression Vanallen2015 cohorts. *P < 0.05, **P < 0.01, ***P < 0.001.

Figure 8A displays the associations between HOXB9 and 47 immune checkpoint genes (ICGs) across different cancer types. These results indicate that HOXB9 expression is linked to multiple ICGs in diverse cancer types. Especially, HOXB9 exhibited a positive correlation with most immune regulators in LIHC, while demonstrating a negative correlation with most immune regulators in READ and COAD. TMB [39] and MIS [40] are novel biomarkers to predict response to immune checkpoint inhibitors. We evaluated the potential of HOXB9 expression as a predictor of immune checkpoint inhibitor (ICI) efficacy by examining its correlation with tumor mutational burden (TMB) and microsatellite instability (MSI) across all tumors in the TCGA dataset. We observed a positive correlation between HOXB9 expression and TMB for BRCA, ESCA, HNSC, LGG, LUAD, LUSC, MESO, PAAD, PRAD, STAD, and THYM but found negative correlations for CESC, KIRC, KIRP, SKCM, and UCEC (Figure 8B). In addition, Figure 8C shows positive associations were discovered in GBM and STAD, and negative correlations were discovered in COAD, READ, THCA, UCEC, and UCS. This study suggests a potential correlation between high HOXB9 levels and immunity in certain tumors, indicating the need for further in-depth research.

### The correlation between HOXB9 expression and immunotherapy response

Anti-PD-L1 antibodies, along with other immune checkpoint inhibitors, have made significant contributions to the cancer immunotherapy [41, 42]. We explored the predictive role of HOXB9 in immunotherapy response according to previously reported cohorts. In the GSE91061 melanoma cohort [34], the relationship between HOXB9 expression and response to anti-PD-1 therapy in melanoma patients suggests that individuals with low HOXB9 expression had better survival rates and longer survival times compared to those with high HOXB9 expression (Figure 8D). Moreover, the HOXB9 high-expression subgroup showed a 0% response rate to anti-PD-1 therapy, while the HOXB9 low-expression subgroup had a response rate of 23.8%. Further, in the Gide2019 melanoma cohort [35], the response rate to anti-CTLA- 4/anti-PD-1 therapy was 25% in high-HOXB9 expression patients, significantly lower than 71.4% in low-HOXB9 expression patients (Figure 8E). Similarly, in the Vanallen2015 cohort [36], our analysis also revealed that patients with low HOXB9 expression had a significantly higher response rate (20.6%) to CTLA-4 blockade therapy compared to patients with high HOXB9 expression, who had a response rate of 0% (Figure 8F). These findings confirm the potential of HOXB9 as a predictor of response to immunotherapy and highlight its potential as a valuable biomarker for cancer immunotherapy.

### Relationship between HOXB9 expression and MMR defects and DNA methyltransferases

**Figure 9:**
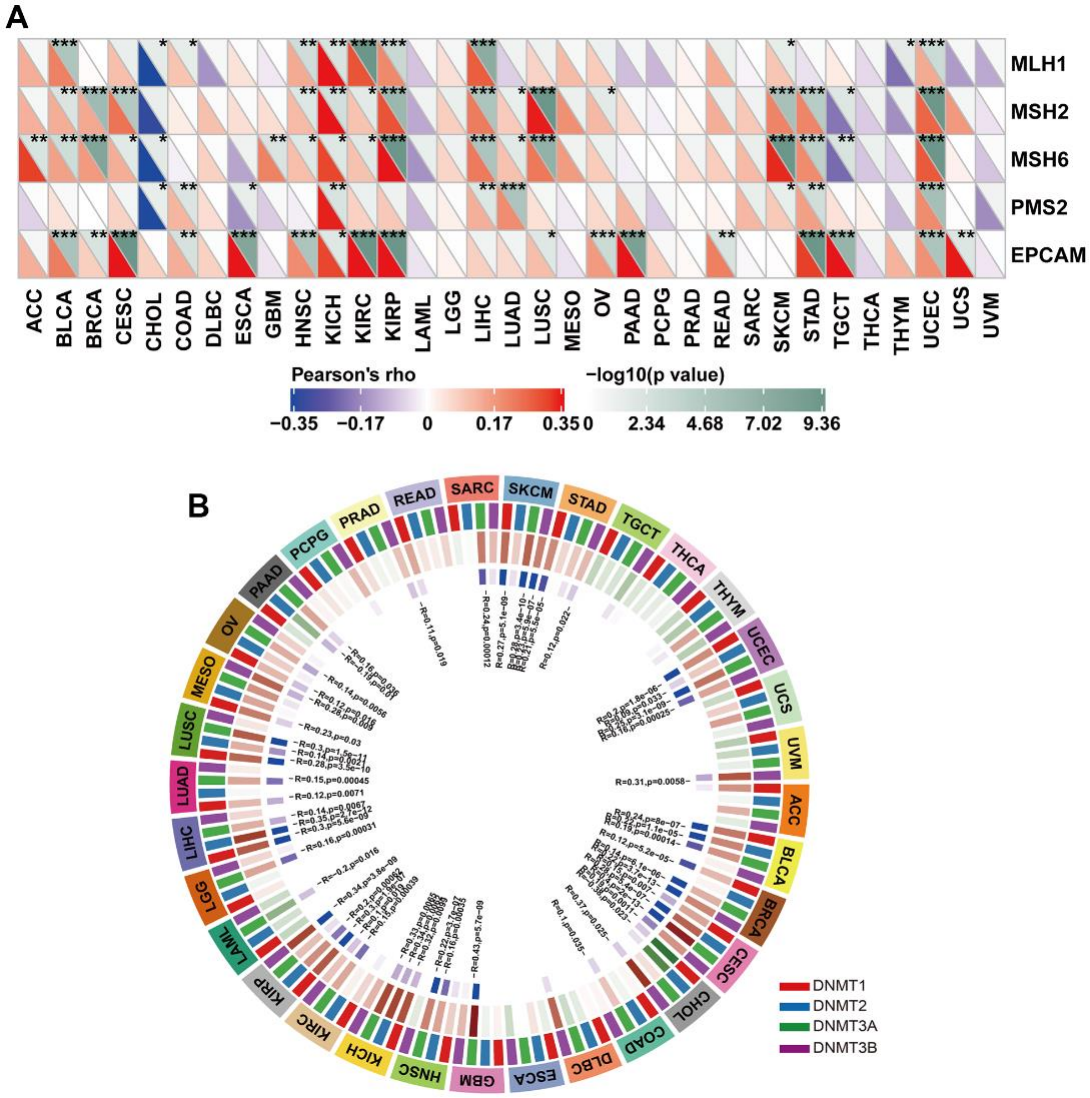
**Correlation between HOXB9 expression and MMR gene mutation and DNA methylation expression.** (**A**) The analysis of HOXB9 expression and five MMR gene expression levels, including MLH1, MSH2, MSH6, PMS2, and EPCAM in pan-cancer. (**B**) The correlation analysis of HOXB9 expression level with four methyltransferase genes (DNMT1, DNMT2, DNMT3A, DNMT3B) in pan-cancer. *P < 0.05, **P < 0.01, ***P < 0.001.

We then investigated the association between HOXB9 gene expression and the mechanisms underlying tumorigenesis, specifically focusing on DNA methylation of genes essential for tumorigenesis and mismatch repair deficiency. Defects in MMR can result in the accumulation of somatic mutations that cannot be repaired, which can contribute to tumorigenesis [43]. We found the five MMR genes were associated with HOXB9 and reached a significant correlation in some cancers (Figure 9A), which may suggest that MMR may have a potential role in regulating tumorigenesis by reducing the rate of DNA replication errors. DNA methylation is an epigenetic mechanism that can significantly decrease the rate of DNA replication errors by regulating gene expression through chromatin structure and DNA conformation modifications [44], and how DNA interacts with proteins [45, 46]. Aberrant DNA methylation patterns played a potential role for this epigenetic modification in tumorigenesis [47]. Therefore, we further evaluated the relationship between HOXB9 and DNA methyltransferases (DNMT1, DNMT2, DNMT3A, and DNMT3B). Interestingly, as for results shown in Figure 9B, expression of HOXB9 was highly correlated with these methyltransferases in a variety of cancers, specifically in SKCM, UCEC, BLCA, BRCA, CESC, GBM, HNSC, KIRP, LIHC, and LUSC. Taken together, this study revealed that HOXB9 might regulate tumorigenesis by affecting DNA repairment and DNA methylation across cancers.

### Knockdown of HOXB9 inhibits cell proliferation, migration, and invasion

**Figure 10:**
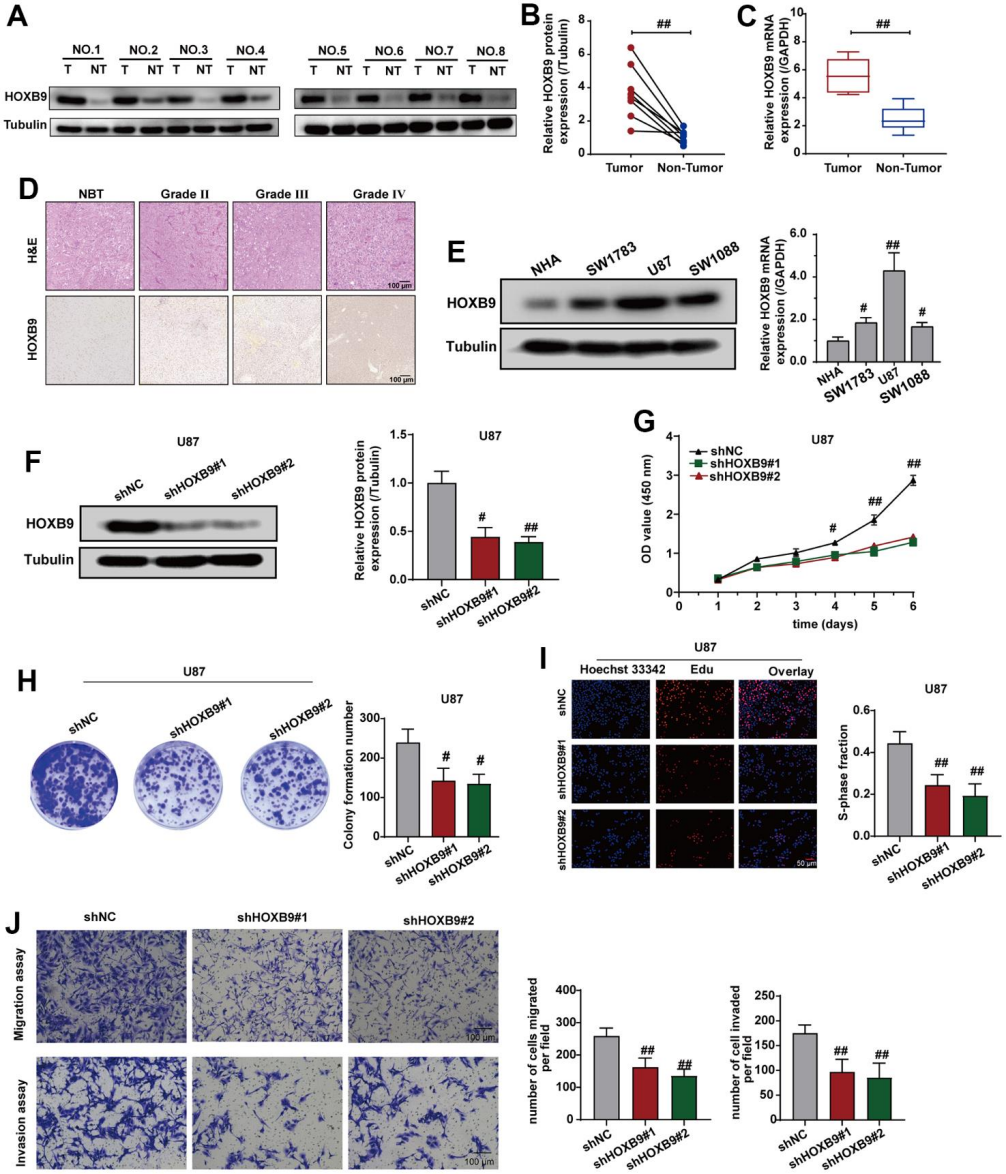
**Knockdown of HOXB9 expression inhibits cells proliferation and invasion.** (**A**) HOXB9 expression levels in GBM tissues and adjacent normal tissues. (**B**, **C**) Quantification analysis of HOXB9 protein and mRNA expression in eight paired GBM tissues and their adjacent normal tissues. (**D**) Representative H&E and IHC staining of HOXB9 in GBM and paired normal tissues (magnification ×200). Scale bar, 100 μm. (**E**) The results of western blotting and qRT-PCR detected the levels of HOXB9 expression in glioma cell lines (SW1783, U87, and SW1088) compared with normal human astrocytes (NHA). (**F**) The HOXB9 expression of the shHOXB9 group is less than that of the shNC group in U87 cells. (**G**–**I**) CCK-8 (**G**), colony formation (**H**), and EdU assays (**I**) were used to detect the proliferation ability in U87 cells transfected with shHOXB9 plasmids. (**J**) Transwell assays detected the migration and invasion ability in U87 cells transfected with shHOXB9 plasmids. ns > 0.05, ^#^*P* < 0.05, ^##^*P* < 0.01.

To validate the protein expression levels of HOXB9 in clinical samples of GBM in comparison to adjacent tissues, we conducted additional experiments using western blotting and qRT-PCR analyses. Figure 10A–10C suggested that the protein and mRNA of HOXB9 were upregulated in GBM samples, consistent with IHC results (Figure 10D). Next, we found that HOXB9 expressions were increased in glioma cell lines (SW1783, U87, and SW1088) compared with normal human astrocytes (NHA) (Figure 10E). Therefore, we downregulated the expression of HOXB9 in U87 cells. Western blotting and qPCR results verified the knockdown status of HOXB9 in U87 cell lines (Figure 10F).

To explore the role of HOXB9 in regulating cell proliferation and invasion, we first assessed the impact of HOXB9 on the proliferative capacity of U87 cells. The results of CCK-8 (Figure 10G), colony formation (Figure 10H), and Edu assays results (Figure 10I) showed that downregulating HOXB9 suppressed cell growth. Consistent with these findings, transwell assays revealed the downregulating HOXB9 expression could suppress the migration and invasion ability in U87 cells (Figure 10J). Given all of that, these data suggested that HOXB9 was overexpressed in GBM tissues, and knockdown of HOXB9 could inhibit proliferation, migration, and invasion in U87 cells.

## DISCUSSION

In recent years, cancer immunotherapy has made significant progress, particularly in the areas of immune checkpoint blockade and chimeric antigen receptor T (CAR-T) cell therapy [48], making cancer patients see the hope for a cure. However, immunotherapy is only effective for some cancers. Immunotherapy has shown great potential in treating cancer, but due to the heterogeneity of the tumor microenvironment in each patient, only a small subset of cancer patients respond favorably to these treatments. This can be attributed to a variety of factors, such as the immune system’s ability to recognize and attack cancer cells, the patient’s overall health status, and the molecular characteristics of the tumor itself. Therefore, developing strategies to improve the response rate to immunotherapy remains a major challenge in the field of cancer research. This study found that HOXB9 is a robust pan-cancer prognostic biomarker that can effectively predict immunotherapy response.

Firstly, to begin with, the expression profile of HOXB9 was evaluated in multiple types of cancer using data from the public databases. The results suggested that HOXB9 was increased in most cancer types. In addition, the GBM samples also showed that HOXB9 expression in GBM tissues was higher than that in adjacent normal tissues, which further confirmed the bioinformatics analysis. In summary, our findings propose that HOXB9 displays abnormal expression across diverse types of cancer, potentially establishing it as a novel cancer biomarker for future diagnostic and prognostic applications.

We further observed the genetic alteration frequency of HOXB9 in different tumors. Most tumors have gene alteration of HOXB9, except LAML, LGG, CHOL, DLBCL, KICH, and TGCT. The frequency of HOXB9 alteration was the highest in UCS, which was about 6%, and BRCA is next at about 5%. We speculated that might be due to the smaller number of patients in the HOXB9-altered group. Therefore, further molecular experimental evidence is required to determine whether HOXB9 alterations play a critical role in the initiation and development of the aforementioned tumors. It is worth noting that HOXB9 was significantly associated with the pathological stages of several tumors, including CESC, HNSC, PAAD, and LIHC, which also showed HOXB9 expression had a forceful association with the degree of tumor infiltrations.

Subsequently, an investigation was conducted to analyze the correlation between HOXB9 and the prognosis of cancer patients. Our findings revealed a strong and consistent correlation between HOXB9 expression levels and cancer patient prognosis across multiple analyses, including OS, DSS, DFI, and PFI. Specifically, elevated HOXB9 expression was identified as a significant risk factor for a considerable proportion of cancer types. These results support prior observations that high HOXB9 expression is associated with poor prognosis in numerous cancers. Furthermore, high expression of transcriptional factor HOXB9 predicts poor prognosis in patients with lung adenocarcinoma [14]. Overall, these results suggest that HOXB9 could serve as an important predictor of cancer patient prognosis.

Treatment methods include cancer vaccines, therapeutic antibodies, and ICIs [49]. Our study was the first to suggest a positive correlation between HOXB9 expression and the extent of infiltration of CAFs, MDSCs, and macrophages in specific types of tumors, which indicates that it is highly likely that HOXB9 impacts the development and prognosis of cancers by modulating the tumor microenvironment. Subsequently, the correlation analysis suggested that HOXB9 is positively correlated with the expression of several immune regulatory genes, especially in LIHC, KICH, and READ. According to the study by Picarda et al., targeting CD276 has led to significant success in the realm of tumor immunotherapy [50]. Furthermore, our results demonstrate a significant correlation between HOXB9 expression and CD276 expression in several cancers, including BLCA, CESC, COAD, GBM, KIRC, KIRP, LAML, LGG, LIHC, LUSC, MESO, OV, PCPG, TGCT, and UVM. We suggest conducting additional research to explore the possible correlation between HOXB9 and CD276.

Earlier research has indicated a strong connection between TMB, MSI, and the effectiveness of immunotherapy [51, 52]. Our study provides initial evidence of a potential association between HOXB9 expression and MSI or TMB in all TCGA tumors. Additionally, our analysis of clinical cohorts with documented responses to PD-1 and CTLA-4 blockade therapy revealed a strong relationship between HOXB9 expression and the effectiveness of immunotherapy. We can effectively predict the response to anti-PD-1/anti-CTLA-4 immunotherapy. Based on these findings, HOXB9 holds great promise as a reliable immunotherapy biomarker for cancer and may have significant clinical implications for cancer treatment. The MMR system consists of several heterodimers as a predictor of tumorigenesis, which can recognize and correct genetic mutations [53]. At the same time, the mutation of the MMR can result in genomic or MSI, leading to tumorigenesis [54]. Our pan-cancer analysis indicates that in the majority of cancers, there is a strong correlation between HOXB9 expression and the mutation levels of MMR genes. Last, our *in vitro* experiments revealed that reducing the expression of HOXB9 significantly inhibited the proliferation and invasive ability of glioma cells. In addition, HOXB9 appears to play a different biological function in different tumors. The DNMT1/RBL2/c-Myc axis is involved in the inhibitory effect of HOXB9 on pancreatic cancer cell proliferation by blocking cell cycle progression [26]. It should be noted that there is some controversy surrounding the role and expression of HOXB9 in gastric and colon cancer. Its function can be complex and dependent on various factors. Some studies have suggested that HOXB9 is downregulated in gastric cancer and colon cancer and is associated with poor clinical outcomes, which suggests it plays an important role as a tumor suppressor gene [15, 55]. The divergent functions of HOXB9 in different types of cancer suggest that the development and progression of cancer are complex processes, and the heterogeneity of cancer tissues should be taken into consideration in specific contexts.

In this study, we systematically analyzed and described the role of HOXB9 in multiple tumors and suggested that HOXB9 may become a valuable tumor biomarker to a great extent. Admittedly, although we collected data from various databases, the present study has some limitations. First, the data of this study were obtained from a large number of tumor and normal samples in multiple databases. Therefore, there may still be some systematic bias because of the limitations of the analytical method. Second, we performed *in vitro* experiments using only glioma cells. *In vivo* and *in vitro* experiments in other types of tumors will significantly increase the persuasiveness of the article. Third, although we elucidated the correlation between HOXB9 expression and tumor immunity, the underlying mechanism remains to be further explored.

## CONCLUSIONS

In conclusion, our study provides a comprehensive analysis of the role of HOXB9 in various cancers and suggests that it has the potential to serve as a prognostic biomarker and a predictor of immunotherapy response. In the clinical perspective, a better understanding of the specific mechanism of HOXB9 in tumorigenesis is aided by the significant correlation observed between HOXB9 and various factors, including prognosis, immune regulation, infiltration of immune cells, tumor microenvironment, tumor mutational burden (TMB), and microsatellite instability (MSI), especially when HOXB9 is expressed at high levels. Based on these findings, it is reasonable to propose that HOXB9 could be a promising target for tumor immunity and a valuable prognostic marker for different types of cancer.

## Supplementary Material

Supplementary Figures

Supplementary Tables 1-3

Supplementary Table 4
