# Supplementary material for: Pan-cancer analysis of Homeobox B9 as a predictor for prognosis and immunotherapy in human tumors
Source: Aging (Albany NY). 2023 Jun 9;15(11):5096–124. doi: 10.18632/aging.204785 (PMC10292867; doi:10.18632/aging.204785)
Supplement: Supplementary Figures [file aging-15-204785-s001.pdf]

SUPPLEMENTARY FIGURES

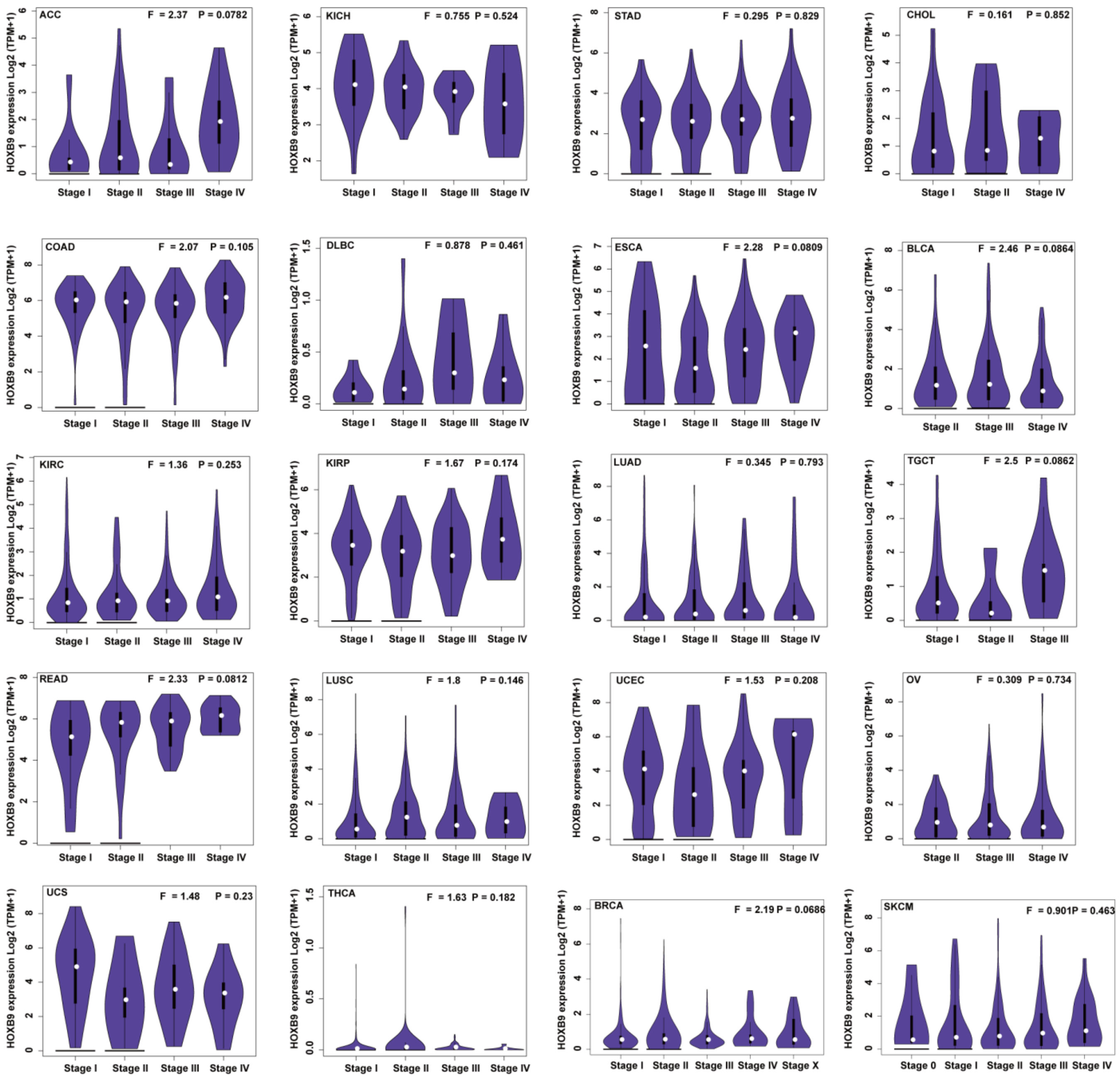

Supplementary Figure 1. Based on the TCGA data, the expression levels of the HOXB9 gene were analyzed by the main pathological stages in 20 cancers.

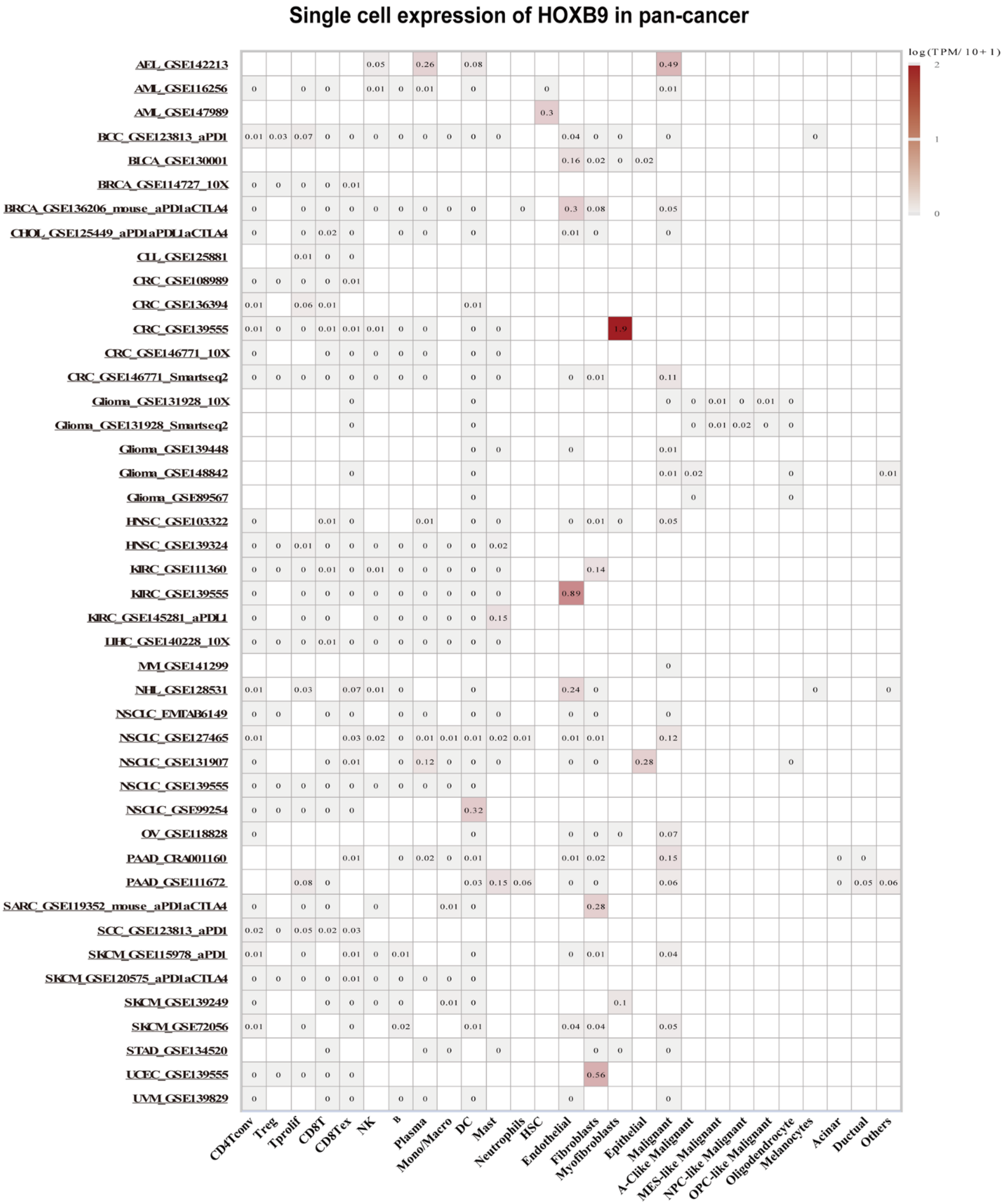

Supplementary Figure 2. HOXB9 expression at the single-cell level in the pan-cancer analysis.

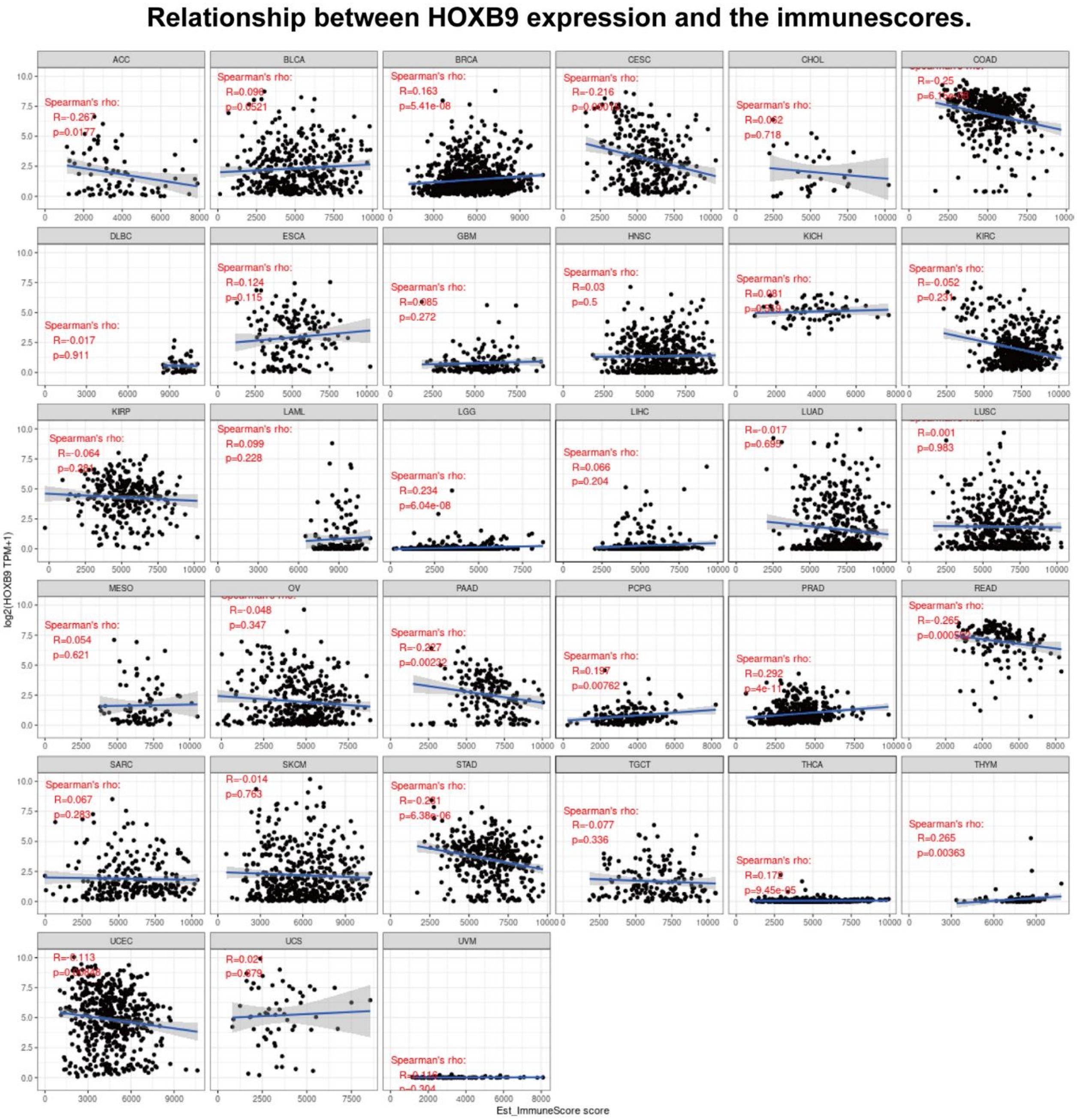

Supplementary Figure 3. Correlation analysis between expression levels of HOXB9 and immunescore in pan-cancer.

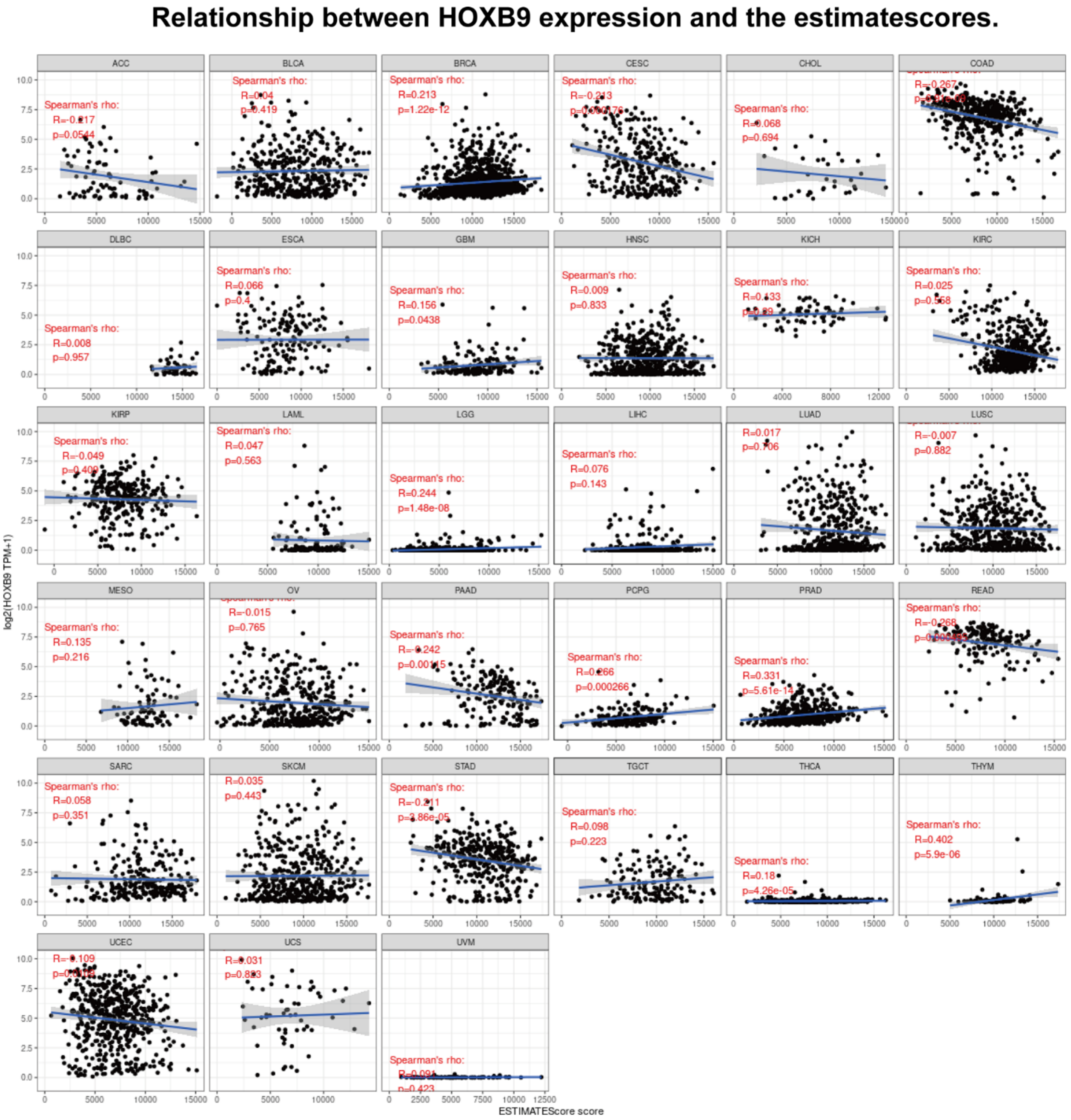

Supplementary Figure 4. Correlation analysis between expression levels of HOXB9 and estimatescore in pan-cancer.

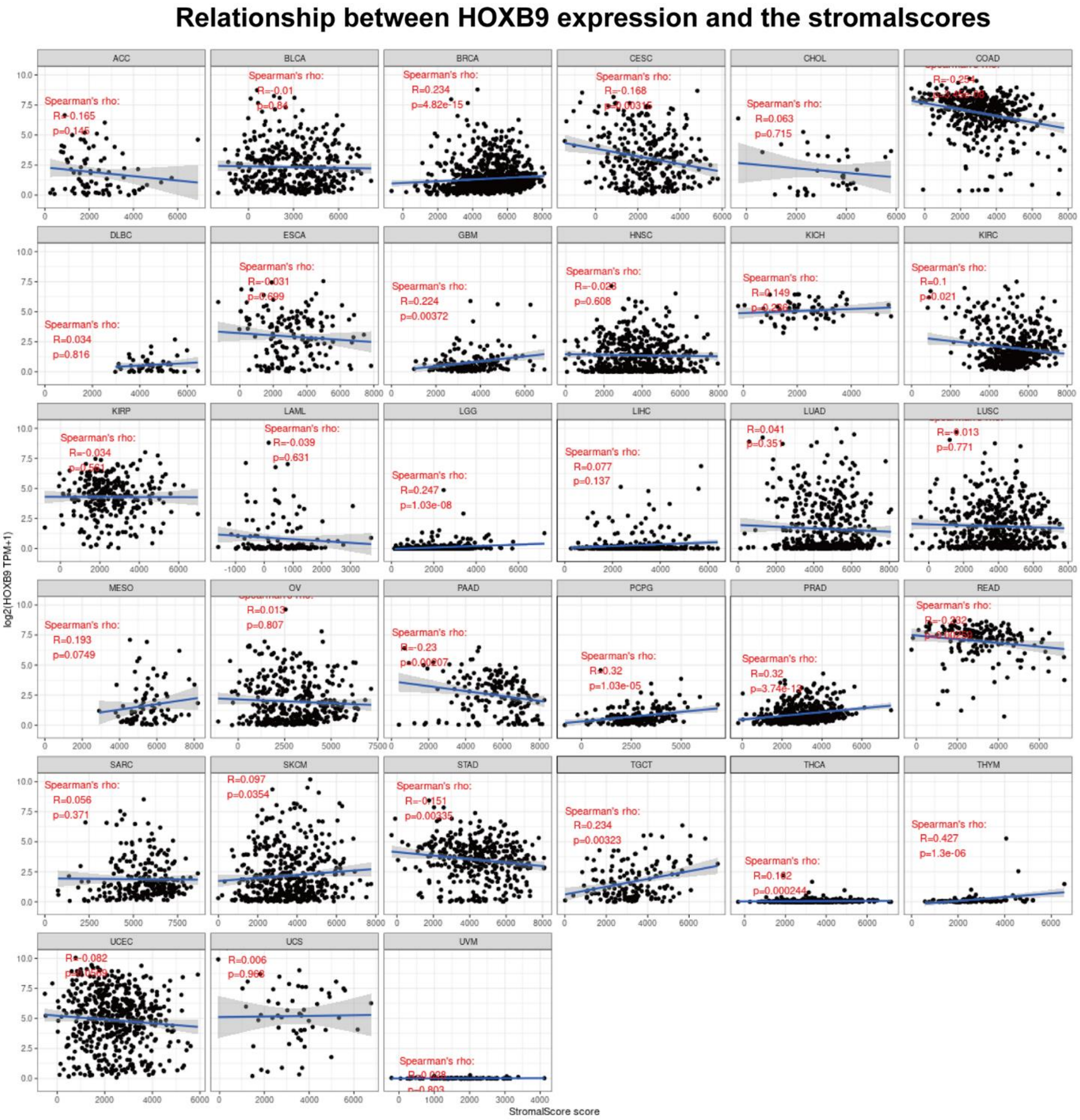

Supplementary Figure 5. Correlation analysis between expression levels of HOXB9 and stromalscore in pan-cancer.

Relationship between HOXB9 levels and neoantigens in pan-cancer

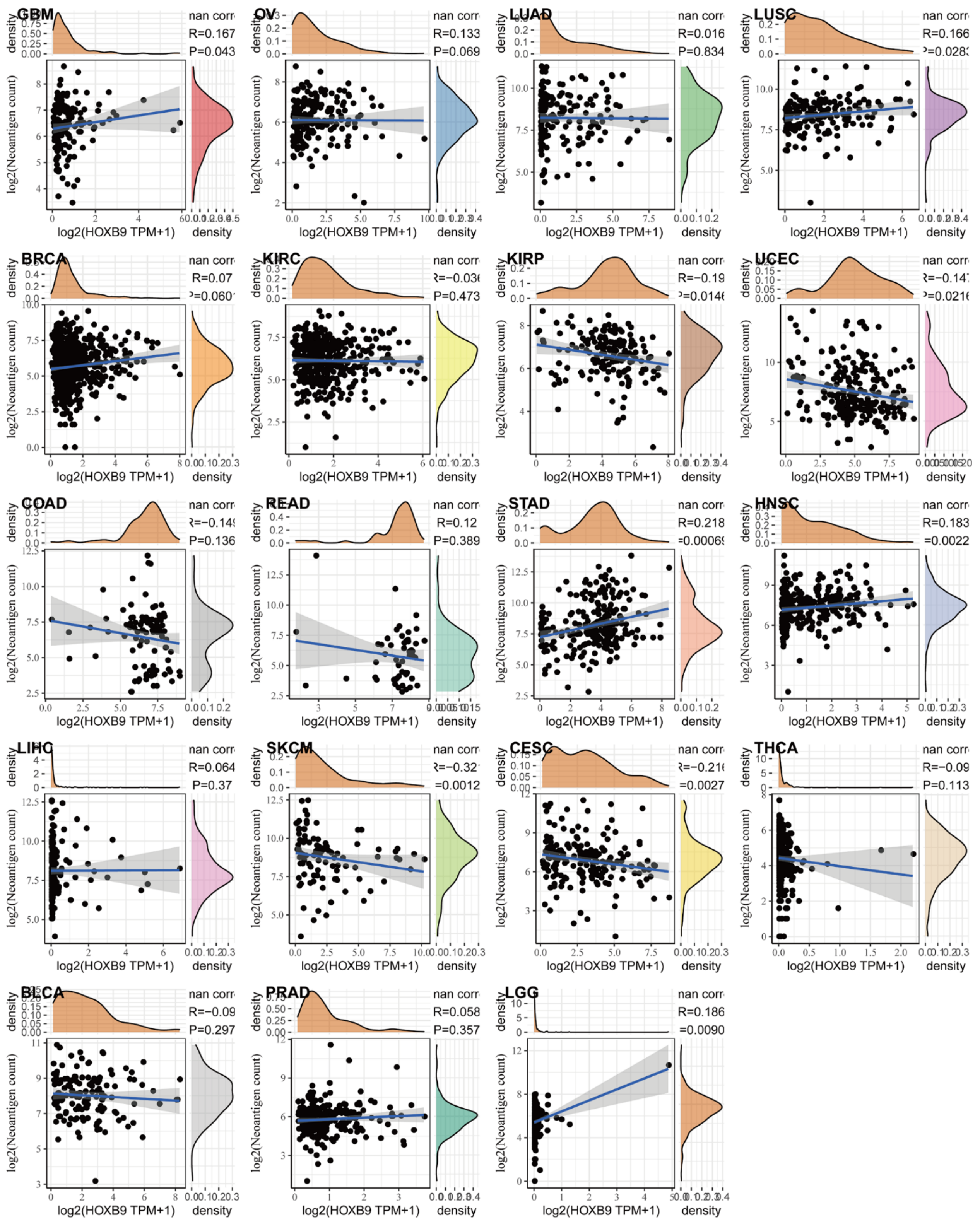

Supplementary Figure 6. Correlation analysis between HOXB9 expression in pan-cancer and the number of tumors neoantigens in pan-cancer.
