# Supplementary material for: Pan-cancer analysis of Homeobox B9 as a predictor for prognosis and immunotherapy in human tumors
Source: Aging (Albany NY). 2023 Jun 9;15(11):5096–124. doi: 10.18632/aging.204785 (PMC10292867; doi:10.18632/aging.204785)
Supplement: Supplementary Tables 1-3 [file aging-15-204785-s002.pdf]

## SUPPLEMENTARY TABLES

**Supplementary Table 1. Abbreviations of cancers.**

| Abbreviations | Full name |
|---|---|
| ACC | Adrenocortical carcinoma |
| BLCA | Bladder Urothelial Carcinoma |
| BRCA | Breast invasive carcinoma |
| CESC | Cervical squamous cell carcinoma and endocervical adenocarcinoma |
| CHOL | Cholangiocarcinoma |
| COAD | Colon adenocarcinoma |
| COADREAD | Colon adenocarcinoma/Rectum adenocarcinoma Esophageal carcinoma |
| DLBC | Lymphoid Neoplasm Diffuse Large B-cell Lymphoma |
| ESCA | Esophageal carcinoma |
| FPPP | FFPE Pilot Phase II |
| GBM | Glioblastoma multiforme |
| GBMLGG | Glioma |
| HNSC | Head and Neck squamous cell carcinoma |
| KICH | Kidney Chromophobe |
| KIPAN | Pan-kidney cohort (KICH+KIRC+KIRP) |
| KIRC | Kidney renal clear cell carcinoma |
| KIRP | Kidney renal papillary cell carcinoma |
| LAML | Acute Myeloid Leukemia |
| LGG | Brain Lower Grade Glioma |
| LIHC | Liver hepatocellular carcinoma |
| LUAD | Lung adenocarcinoma |
| LUSC | Lung squamous cell carcinoma |
| MESO | Mesothelioma |
| OV | Ovarian serous cystadenocarcinoma |
| PAAD | Pancreatic adenocarcinoma |
| PCPG | Pheochromocytoma and Paraganglioma |
| PRAD | Prostate adenocarcinoma |
| READ | Rectum adenocarcinoma |
| SARC | Sarcoma |
| STAD | Stomach adenocarcinoma |
| SKCM | Skin Cutaneous Melanoma |
| STES | Stomach and Esophageal carcinoma |
| TGCT | Testicular Germ Cell Tumors |
| THCA | Thyroid carcinoma |
| THYM | Thymoma |
| UCEC | Uterine Corpus Endometrial Carcinoma |
| UCS | Uterine Carcinosarcoma |
| UVM | Uveal Melanoma |
| OS | Osteosarcoma |
| ALL | Acute Lymphoblastic Leukemia |

**Supplementary Table 2. The relevant reagents used in this article.**

| REAGENT | SOURCE | IDENTIFIER |
|---|---|---|
| Rabbit Anti-HOXB9 | SANTA CRUZ BIOTECHNOLOGY | Cat# sc-130377 |
| Rabbit Anti- Alpha Tubulin | Proteintech | Cat# 11224-1-AP |
| DAPI | Sigma-Aldrich | Cat# D9542 |
| Crystal violet | Solarbio | Cat# C8470 |
| Fetal bovine serum | Gibco | Cat# 10099141 |
| PBS | ZSGB-BIO | Cat# ZLI 9061 |
| Cell Counting Kit-8 Kit | Beyotime Institute of Biotechnology | Cat#C0037 |
| Edu Kit | Ribobio | Cat#C10310 |
| BCA protein assay kit | Beyotime | Cat#P0012 |

**Supplementary Table 3. Primer sequences for qPCR.**

| Name | Sequences |
|---|---|
| **Primers for real-time PCR:** | |
| HOXB9 sence: | 5’-CCATTTCTGGGACGCTTAGCA-3′ |
| HOXB9 antisence: | 5’-TGTAAGGGTGGTAGACGGACG-3′ |
| GAPDH sence: | 5’-GGTCGGAGTCAACGGATTTGGTCG-3′ |
| GAPDH antisence: | 5’-CCTCCGACGCCTGCTTCACCAC-3′ |
